# Custom-made acetabular implants for severe acetabular bone defects: implant evolution, mid-term clinical and radiographic outcomes, and complications in 154 consecutive patients over 18 years

**DOI:** 10.1186/s42836-026-00425-3

**Published:** 2026-09-02

**Authors:** Mari Babasiz, Alberto Alfieri Zellner, Tamara Babasiz, Robert Ossendorff, Frank Sebastian Fröschen, Andreas Christian Strauss, Max Jaenisch, Dieter Christian Wirtz

**Affiliations:** 1 Department for Orthopaedic and Trauma Surgery, University Hospital Bonn, Venusberg Campus 1, 53127 Bonn, Germany; 2 Department for Orthopaedic and Trauma Surgery, University Hospital Cologne, Kerpener Straße 62, 50937 Cologne, Germany

**Keywords:** Custom-made acetabular implant, Revision total hip arthroplasty, Acetabular defect, Pelvic discontinuity, Patient-specific implant

## Abstract

**Background:**

Custom-made acetabular components (CMAC) represent an important treatment option for complex acetabular defects where standard implants are not sufficient. Over the years, substantial advances in implant design and materials have improved stability, function, and longevity of implants. However, evidence on implant survivorship and design evolution in custom-made acetabular reconstruction remains limited.

**Methods:**

In this retrospective single-center study, 154 consecutive patients treated with a CMAC between October 2007 and June 2025 were analysed. All reconstructions were performed for severe acetabular defects classified as Paprosky type 3A (*n* = 85) or 3B (*n* = 69), corresponding to the Acetabular Defect Classification (ADC) type 3C (*n* = 85) and ADC type 4 (*n* = 69) with pelvic discontinuity (4A *n* = 14, 4B *n* = 28, 4C *n* = 27). Demographic data, implant design characteristics, and surgical technique were recorded. Clinical outcomes were assessed using the Oxford Hip Score (OHS), all complications were recorded, and radiographic evaluation was performed.

**Results:**

The mean follow-up was 34.82 months (± 26.2 range, 6.2–168 months). Paired preoperative and postoperative OHS improved from 21.69 ± 5.59 to 30.93 ± 5.26, corresponding to a mean improvement of 9.23 points (*p* < 0.0001).

Kaplan–Meier estimated survivorship at 3 and 5 years was 0.85 (SE = 0.04) and 0.78 (SE = 0.05) for explantation and 0.64 (SE = 0.04) and 0.58 (SE = 0.05) for reoperation for any reason. Postoperative periprosthetic infection (11.0%) and dislocation (10.4%) were the most frequent complications. In an exploratory multivariable Cox model, flange design was the only covariate associated with explantation (HR 17.70, 95% CI 4.78–65.52; *p* < 0.001).

**Conclusions:**

This 18-year single-centre experience with 154 CMAC demonstrates the mid-term clinical reliability of an individualized approach to complex acetabular reconstruction. Postoperative periprosthetic infection (11.0%) and dislocation (10.4%) remained relevant complications. The design transition from multiflange to monoflange constructs represents the principal evolution of this experience and warrants dedicated comparative evaluation.

## Background

The treatment of massive acetabular bone loss has remained a major challenge in orthopedic reconstruction [1]. Large defects following hip arthroplasty removal often exceed the reconstructive capabilities of modular implants and cages [2]. For such cases, custom-made acetabular implants may represent a valuable solution for filling the acetabular defect and reconstructing the center of rotation [3]. Classification systems guide reconstruction in acetabular bone loss. While the Paprosky classification remains widely used [4], it may not fully reflect complexity in severe acetabular bone defects, especially in cases with pelvic discontinuity. To address this gap, the Acetabular Defect Classification (ADC) has been proposed as a more therapy-oriented framework based on rim integrity, columnar support, and discontinuity severity [5].

Recent literature demonstrated that the implantation of custom-made acetabular implants may yield beneficial postoperative outcomes [6, 7]. Although these results are encouraging, serious challenges remain [8, 9]. In complex acetabular revision cases, the altered anatomy creates a highly challenging surgical environment. As a result, there is a substantially increased risk of complications [8, 9]. Based on 18 years of experience with custom-made acetabular components (CMAC), we developed a distinct surgical philosophy regarding indications, fixation principles, and implant design, derived from longitudinal outcomes and intraoperative experiences. From our perspective, three primary indications justify the use of a custom-made acetabular replacement: (1) a large cranio-caudal defect exceeding 100 mm; (2) destruction of both the anterior and posterior columns; and (3) pelvic discontinuity.

**Fig. 1:**
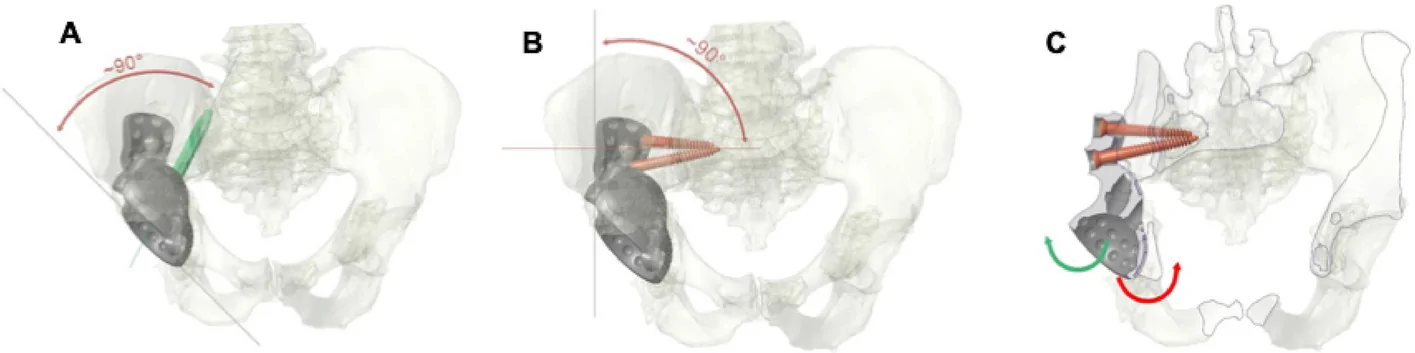
The 90°−90° cranial fixation concept in custom-made monoflange acetabular reconstruction. **A** Three-dimensional preoperative planning of a custom-made monoflange acetabular component. The central iliac peg is aligned at 90° to the acetabular inclination entry axis. **B** Orientation of the trans-iliosacral screws at 90° relative to the iliac flange (**C**) Schematic illustration of forces acting on the construct, demonstrating vectors of potential medial and lateral tilting. The images were kindly provided by PETER BREHM GmbH

**Fig. 2:**
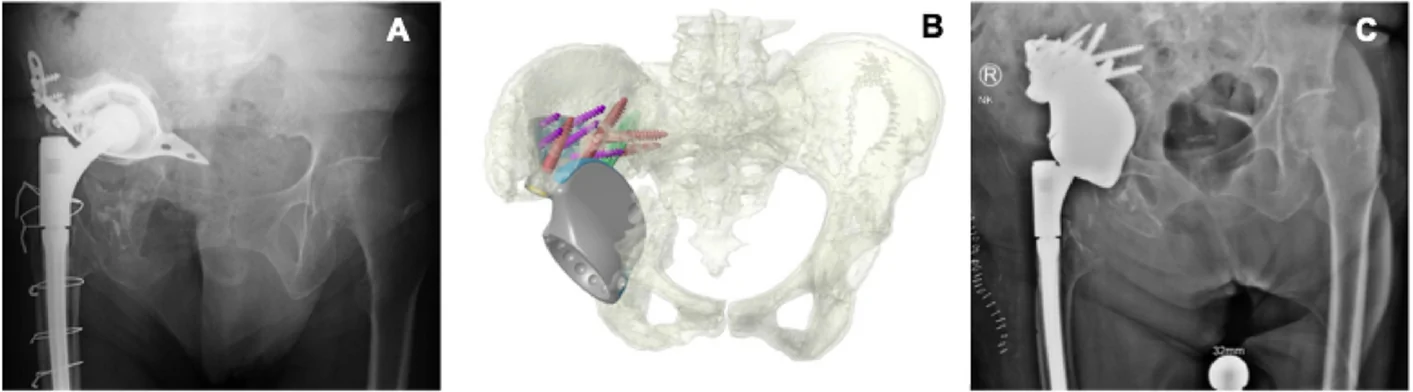
Preoperative three-dimensional planning and pre- and postoperative radiographic appearance. **A** Preoperative anterior–posterior pelvic radiograph showing a loosened and migrated revision acetabular component with intrapelvic protrusion (ADC Type 4 C). **B** Three-dimensional preoperative planning of a CMAC illustrating the biomechanical rationale of the 90°–90° fixation concept at the os ilium. The image were kindly provided by PETER BREHM GmbH. **C** Postoperative radiograph demonstrating the planned and implanted custom-made monoflange acetabular component using a cranial fixation strategy. Fixation is achieved by an iliac flange with angle-stable screws, a central iliac peg, and two trans-iliosacral screws applied according to the planned 90°–90° fixation technique

Our reconstructive strategy originally relied on triflange constructs with fixation at the os ilium, os pubis, and os ischium. Although this three-point fixation concept provided theoretical stability, it frequently required extensive exposure near the iliac vessels in the pubic region and carried a risk of sciatic nerve injury in the ischial area. With increasing clinical experience, it became evident that in severe acetabular defects, osteolysis of the inferior hemipelvis often precludes reliable fixation in the pubic and ischial regions. Consequently, we redefined our indication for inferior fixation: screws in the os pubis or os ischium are only considered when the pelvic ring is intact, no osteolysis in the os pubis and os ischium is present, and the defect corresponds to ADC type 3. In these cases, a screw placed in these regions may support accurate positioning of the acetabular implant and facilitate optimal restoration of the center of rotation. However, in the presence of pelvic discontinuity (ADC type 4), fixation to the os pubis or os ischium should not be pursued, as the bone stock is typically deteriorated and offers no biomechanical advantage. Thus, we adopted a monoflange-based fixation philosophy, centered on stable cranial anchorage as the key determinant of mechanical control. Implantation has been optimized using a standardized 90°−90° fixation concept (Figs. 1 and 2), defined by an orthogonal screw orientation, in which the trans-iliosacral screws are positioned at a 90° angle relative to the iliac flange, while the iliac peg is aligned 90° to the planned acetabular inclination. By providing stable cranial and iliosacral fixation, the 90°−90° configuration effectively counteracts medial and lateral tilting of the acetabular implant and prevents instability at the iliosacral joint. Fixation concentrated exclusively within the superior ilium and the sacroiliac region, areas with consistent structurally reliable bone stock, establishes a superior load-bearing system capable of resisting axial, shear, and rotational loading. This strategy does not aim to restore osseous continuity across the pelvic discontinuity, but rather is intended to neutralize the discontinuity biomechanically by achieving stability through superior fixation, allowing for secure implant seating despite the persistent separation of the hemipelvis.

Our philosophy, based on nearly two decades of operative experience, has shaped our transition away from inferior fixation strategies and towards a cranial-dominant, orthogonal reconstruction concept that maximizes primary stability. This article presents our experience with 154 CMAC and the effects of an evolving surgical strategy and implant-design optimization on clinical, radiographic, and complication outcomes. The cranial-dominant fixation concept reflects our longitudinal clinical rationale rather than a biomechanically validated principle and, absent direct comparison with three-column fixation or biomechanical testing, remains a hypothesis for future confirmation.

## Materials and methods

### Study design

The present retrospective, single-center study investigated all patients who underwent revision hip arthroplasty, including implantation of a custom-made acetabular implant between October 2007 and June 2025. Inclusion criteria comprised (1) reconstruction using a patient-specific, 3D-printed acetabular implant, (2) adequate clinical documentation to assess clinical and radiological outcomes, and (3) a minimal clinical and radiological follow-up of at least 6 months. Exclusion criteria were (1) reconstructions performed for primary tumor resection or metastasis, (2) acetabular reconstruction using standard hemispherical components, cages, augments, or cup-cage constructs, and (3) insufficient follow-up data. We conducted this study in compliance with the principles of the Declaration of Helsinki. The study was approved by the local institutional review board (2025-249-BO, 09.07.2025).

Survivorship was analyzed at the patient level. Each of the 154 patients contributed one index CMAC reconstruction. All acetabular defects were classified according to the Paprosky classification [4] and the ADC [5]. Pelvic discontinuity was defined as an ADC type 4 defect established on preoperative computed tomography and confirmed intraoperatively. All 69 Paprosky type 3B defects were classified as ADC type 4 and displayed pelvic discontinuity.

Outcome measurements were the implant survivorship with explantation as the primary endpoint [10], survival free from reoperation for any cause as a secondary endpoint, preoperative and postoperative OHS, operation-specific complications (dislocation, infection, neurovascular injury, hematoma), and radiographic evidence for implant osseointegration. Clinical data were obtained from standardized routine follow-up examinations. Standardized anteroposterior pelvic and lateral radiographs obtained at predefined postoperative intervals were used to assess implant integration.

### Statistical analysis

Data were collected using Microsoft Excel 2024 (Microsoft Corporation, Redmond, WA, USA). Statistical analyses were performed with SPSS Statistics version 31 for Macintosh (IBM Corp., Armonk, NY, USA). Descriptive statistics included calculation of arithmetic means and standard deviations, with continuous variables reported as mean (M) ± standard deviation (SD). An alpha level of 0.05 was considered statistically significant. Normality of continuous variables was assessed using the Shapiro–Wilk test. Pre- and postoperative OHS were compared using the Wilcoxon signed-rank test for paired samples. For normally distributed data, group comparisons were performed using an unpaired two-tailed t-test, whereas non-normally distributed variables were analyzed using the Mann–Whitney U test. Ordinal variables were summarized as absolute frequencies and percentages, and group differences were assessed using Pearson’s chi-squared test. Survival analysis was estimated using Kaplan–Meier analysis with patients censored at their last follow-up and numbers at risk reported at predefined intervals. Flange design was entered into the multivariable Cox proportional-hazards model as a binary covariate. Figures were generated using GraphPad Prism version 10 (GraphPad Software, San Diego, CA, USA).

### Implant design

**Fig. 3:**
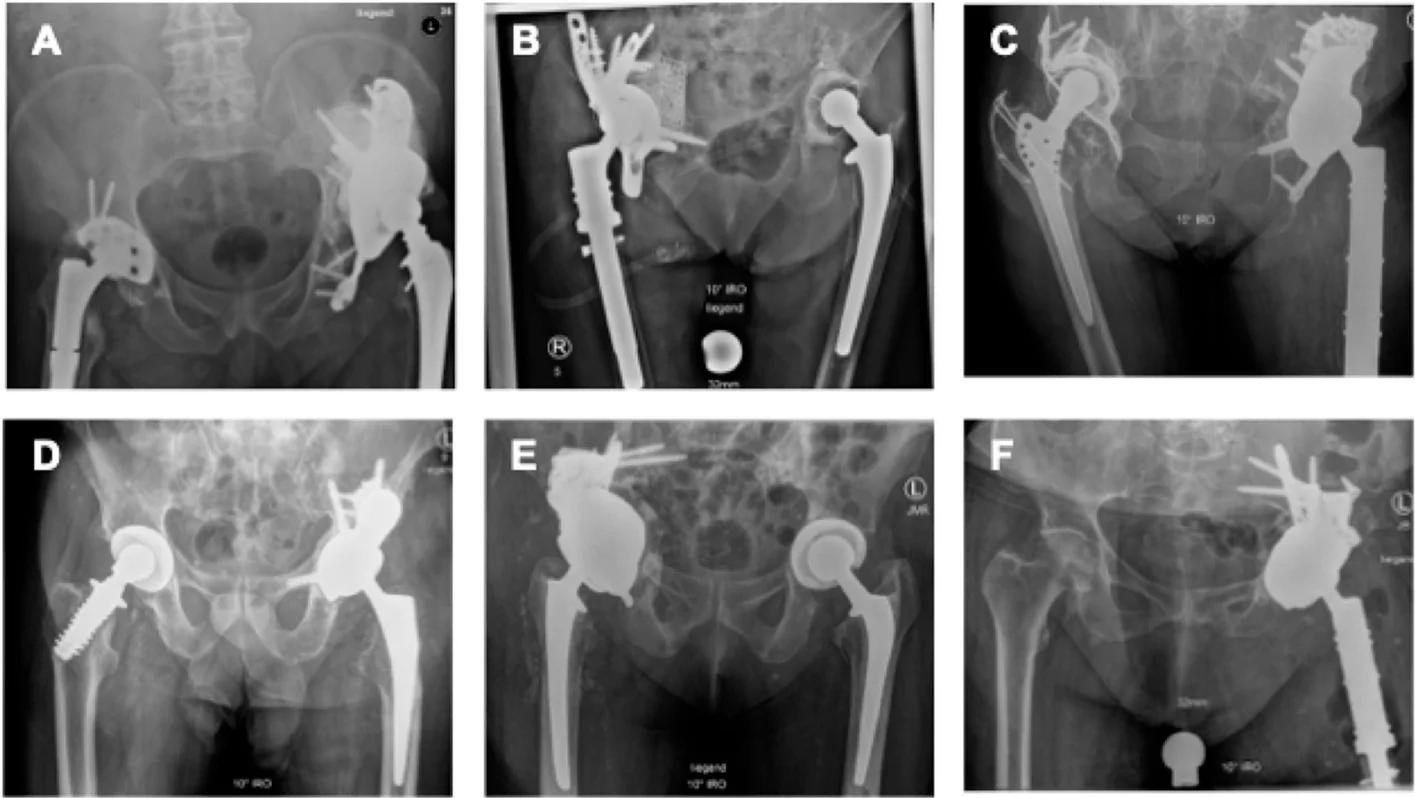
Anteroposterior pelvic radiographs illustrating key stages of custom-made acetabular reconstruction. **A** Left-sided implantation (2008) of a CMAC for severe acetabular bone loss due to aseptic loosening (ADC type 3 C), with cement augmentation of the large cavitary defect. **B** Right-sided triflange implantation (2012) with medial acetabular support provided by a titanium mesh to prevent cement extrusion during augmentation of the medial defect. **C** Left-sided reimplantation (2018) using a biflange component with pubic screw following multistage procedure because of periprosthetic infection. **D** Left-sided monoflange reimplantation (2018) secured with angular-stable flange screws and multiple pelvic 8-mm screws for enhanced fixation in compromised bone (ADC type 3 C). **E** Right-sided monoflange implant (2021) with a 10-mm iliac peg and two transiliosacral screws as a further step in implant evolution (ADC type 4 C). **F** Right-sided monoflange reimplantation (2025) as the final step in the treatment of a pelvic discontinuity following septic loosening. Reconstruction was achieved using a 10-mm iliac peg and two 8-mm trans-iliosacral screws

The implant design has undergone substantial evolution over the past two decades. Initially, reconstruction was performed using triflange or biflange designs with a pubical screw, providing three-point fixation to the ilium, ischium, and pubis. Over time, this concept was refined to a monoflange design, focusing fixation primarily on the ilium while abandoning screws, flanges, and hooks in the pubic and ischial regions, due to limited bone quality and higher risk of sciatic nerve injury and vascular complications during surgical exposure and soft tissue dissection. Based on these observations, we systematically reassessed our surgical and biomechanical strategy and progressively refined our implant philosophy (Fig. 3). As a result of this process, our current philosophy is centered on a monoflange-based implant design with cranial fixation in the ilium as the primary load-bearing structure. Central anchorage is achieved using a 10-mm iliac peg and, if possible, additional 8-mm iliac screws. The central iliac peg should be inserted to a maximal depth with angular-stable blocking providing high primary stability; its surface is roughened and macroporous to promote osseointegration. If using additional 8-mm iliac screws, these should also be angular-stable blocked and placed as long as possible within the os ilium to optimize force transfer. A single iliac flange serves as the main structural interface, while optional additional fixation into the pubis or ischium is reserved for cases with sufficient local bone stock. Subsequent innovations included the introduction of macroporous, 3D-printed titanium acetabular back side surfaces, replacing cement or bone grafts as defect-filling materials and promoting enhanced osseointegration of the implant [11, 12].

Additionally, in selected acetabular defect situations with sacroiliac instability or pelvic discontinuity, two supplementary trans-iliosacral screws of 8-mm diameter can be used. This technique represents the most recent stage of our implant evolution. The decision is made individually for each reconstruction during the preoperative planning discussion between the surgeon and the design engineer. These screws should be cannulated, allowing precise intraoperative placement using the Seldinger technique [13], and should also be angular-stable blocked, providing high structural implant integrity. In cases with pelvic discontinuity with severe bone defects, trans-iliosacral fixation plays a key role in neutralizing varus–valgus forces, preventing medial and lateral tilting of the acetabular component, and reducing the risk of sacroiliac joint destabilization (Figs. 1 and 2).

### Preoperative planning and surgical procedure

All surgical procedures were performed by a senior orthopedic surgeon. The surgical treatment followed a standardized two- or multi- stage protocol. Firstly, the existing acetabular component was explanted. In cases of aseptic revision, the femoral stem was retained in situ if it demonstrated satisfactory fixation and stability. After removal of the acetabular component and intraoperative confirmation that the extent of bone loss required a custom-made acetabular implant, a thin-slice computed tomography (CT) scan of the pelvis was obtained to capture the postoperative defect morphology with high spatial resolution. The CT data were subsequently transferred to the respective manufacturing companies. In our study, implant design was performed in close collaboration with Peter Brehm (Germany), Implantcast (Germany), and Lima Corporate (Italy). The implant geometry, fixation strategy, and contact surfaces were tailored to each patient’s anatomy and defect configuration.

Based on the imaging dataset and the documented intraoperative defect characteristics, the engineers generated a patient-specific 3D implant design. The proposed construct, including flange geometry, screw trajectories, and areas of metal augmentation, was reviewed by the operating surgeon to ensure anatomical accuracy and feasibility. After the surgeon’s approval, the final implant was produced. Modern custom-made acetabular implants are typically manufactured from Ti-6Al-4 V (medical-grade titanium alloy) using additive manufacturing techniques such as electron-beam melting (EBM) or selective laser melting (SLM) [14]. These allow complex, patient-specific geometries and integrated porous lattices [15]. The workflow takes approximately 6–12 weeks and requires close collaboration between the surgical and engineering teams [15, 16]. Following the production period, the patient underwent reimplantation in a second operation. The definitive CMAC was implanted using standard exposure and fixation techniques appropriate to the level of acetabular bone loss and pelvic stability. In recent years, an additional patient-specific sterile 3D-printed pelvic model was generated and brought to the operating room to assist with intraoperative orientation, identification of key anatomical landmarks, and provide both virtual and haptic confirmation of implant positioning and optimal screw placement.

### Clinical and radiological evaluation

Implant failure was defined as explantation of the acetabular component for any reason (primary endpoint), and reoperation for any cause, regardless of whether the component was removed, was assessed as a secondary endpoint. In addition, postoperative complications not leading to implant removal were recorded, including dislocation managed with closed reduction and wound-related revision procedures. Implant survival was assessed using Kaplan–Meier survival analysis. Clinical outcomes were evaluated using the OHS, a validated patient-reported outcome measure assessing pain and functional ability in activities of daily living following hip surgery [17]. The score ranges from 0 to 48, with higher scores indicating better hip function. The OHS was recorded preoperatively at the time of surgical consent and postoperatively at the latest available follow-up visit. Osseointegration of the CMAC was assessed using the five signs of Moore et al. [18] (absence of radiolucent lines, presence of a superolateral buttress, medial stress-shielding, radial trabeculae, inferomedial buttress), with three or more signs classified as osseointegrated. Radiographic failure was defined as migration > 5 mm, inclination change > 5°, progressive radiolucent lines, or hardware fracture. All radiographs were assessed independently by two observers experienced in revision arthroplasty, blinded to clinical outcome, with discrepancies resolved by consensus. As radiographs were read in consensus, formal interobserver reliability was not calculated.

## Results

A total of 154 patients who underwent surgical treatment with implantation of a CMAC were included in this retrospective study. All patients had a severe acetabular bone loss of at least Paprosky Type 3A or at least Acetabular defect classification type ADC 3 C [4, 5]. The cohort consisted of 48 males (31.2%) and 106 females (68.8%). The mean follow-up was 34.82 months (± 26.2 range, 6.2–168 months). Baseline demographic and clinical characteristics of the cohort are summarized in Table 1. The operation was performed via a lateral, transgluteal (*n* = 150; 97.4%) or posterolateral (*n* = 4; 2.6%) approach.

**Table 1:** Patient demographics, defect classification, implant characteristics and perioperative Data (*n* = 154)

**Demographics**	Mean ± SD	*n* (%)
Number of patients		154 (100%)
Age (years)	70.36 ± 13.68	154 (100%)
Body Mass Index (kg/m2)	28.25 ± 7.1	154 (100%)
Sex (male/female)		48 (31.2)/106 (68.8)
Side (right/left)		78 (50.6)/76 (49.4)
ASA II		113 (73.4%)
ASA III		34 (22.1%)
ASA IV		7 (4.5%)
**Defect classification**		
Paprosky type 3A/3B		85 (55.2)/69 (44.8)
ADC type 3C		85 (55.2)
ADC type 4A		14 (9.1)
ADC type 4B		28 (18.2)
ADC type 4C		27 (17.5)
Pelvic discontinuity (ADC type 4)		69 (44.8%)
**Indication**		
Aseptic		80 (51.9%)
Septic		74 (48.1%)
**Implant characteristics**		
Flange design, monoflange		101 (65.6)
Flange design, biflange		44 (28.6)
Flange design, triflange		9 (5.8)
Dual mobility bearing, yes/no		96 (62.3)/58 (37.7)
**Perioperative and follow-up data**		
Operative time (min)	258.2 ± 93.6	154 (100%)
Hospital stay (days)	23.5 ± 9.5	154 (100%)
Follow-up (months)	34.82 ± 26.2 (range, 6.2–168)	154 (100%)

Values are mean ± standard deviation or *n* (%). Percentages refer to the total cohort of 154 patients. ADC = Acetabular Defect Classification; ASA = American Society of Anesthesiologists physical status classification. Pelvic discontinuity corresponds to ADC type 4 and was established on preoperative computed tomography and confirmed intraoperatively; the Paprosky grade and the presence of pelvic discontinuity were assessed independently

**Fig. 4:**
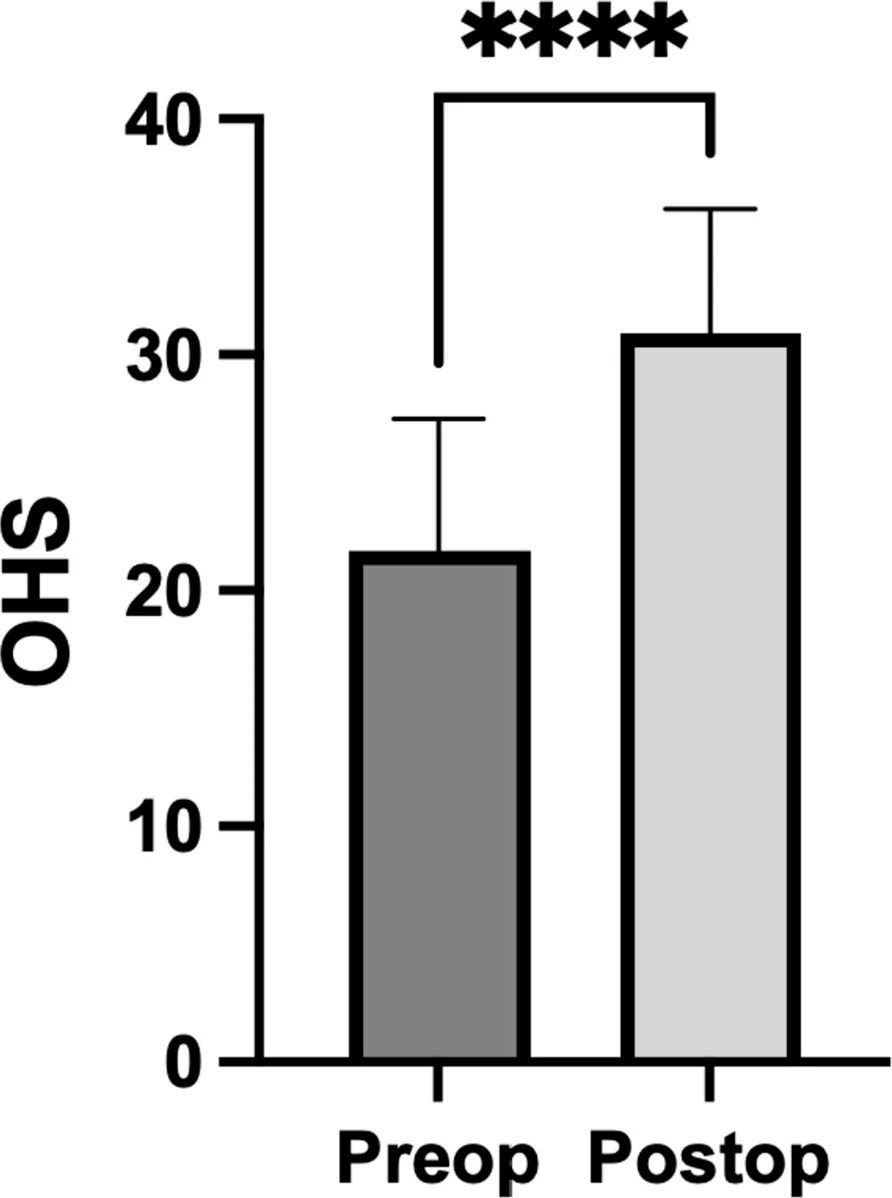
Oxford Hip Score before surgery and at latest follow-up in 147 patients. The mean preoperative score was 21.69 ± 5.59, and the mean postoperative score was 30.93 ± 5.26, corresponding to a mean improvement of 9.23 points. Comparison by Wilcoxon signed-rank test for paired samples, exact two-tailed *p* < 0.0001

Dual mobility cups were not routinely used as standard, especially in the early years. Postoperative dislocation occurred in 10 of 96 patients (10.4%) with a dual mobility construct and in 6 of 58 patients (10.3%) without dual mobility. There was no significant difference between groups based on the chi-square test (χ^2^ = 0.1005, df = 1; *p* = 0.7512). The mean inclination angle in the cohort was 46.44° (SD ± 6.43). Paired preoperative and postoperative OHS were available for 147 patients. The score improved from 21.69 ± 5.59 preoperatively to 30.93 ± 5.26 at the latest follow-up, corresponding to a mean improvement of 9.23 points (*p* < 0.0001; Fig. 4).

Because of the patient-specific implant design, all cases were managed using staged surgical strategies, including two-stage procedures (*n* = 103) and multiple-stage procedures (*n* = 51). The indication for the index custom reconstruction was a previous septic failure in 74 of 154 patients (48.1%). Among these 74 infection cases, *Staphylococcus epidermidis* was the most frequently isolated organism, identified in 36 patients (48.6%), followed by *Staphylococcus aureus* in 14 patients (18.9%) and *Enterococcus faecalis* in 13 patients (17.6%). Twelve of the 74 patients (16.2%) demonstrated polymicrobial infection with more than one pathogen isolated at the time of explantation. All patients received pathogen-directed antibiotic therapy for a total of at least six weeks, consisting of intravenous administration for 14 days followed by oral therapy for four weeks.

There was no significant difference in the risk of implant removal between septic and aseptic indications for revision (χ^2^ = 0.1821, df = 1; *p* = 0.6696). Similarly, the overall revision rate did not differ significantly between groups as assessed by the chi-square test (χ^2^ = 1.332, df = 1; *p* = 0.2485). A total of 19 patients required explantation of the CMAC for any cause. Of these, 9 were explanted for periprosthetic joint infection and 10 for aseptic or mechanical reasons. Of these 19 patients, 4 patients were able to undergo reimplantation with a new custom-made acetabular reconstruction after an interval period, during which infection control or soft-tissue recovery was achieved.

Among all 154 patients, the most common postoperative complication was periprosthetic joint infection, occurring in 17 patients (11.0%). Of these 17 postoperative infections, 10 occurred in patients whose index reconstruction had been performed for a septic indication, whereas 7 occurred in patients revised for aseptic failure and represent new infections. In eight of these 17 patients (47.1%), infection eradication was achieved by surgical debridement, antibiotics, and implant retention (DAIR) with exchange of the mobile components, whereas nine patients (52.9%) required implant removal with conversion to a Girdlestone situation. Dislocation was the second most frequent complication, occurring in 16 patients (10.4%), of whom six (37.5%) were successfully managed with closed reduction without the need for revision surgery or implant modification during follow-up. Intraoperative pelvic fractures occurred in 3 cases (1.9%). One patient (0.6%) sustained a postoperative sciatic nerve palsy. Postoperative hematoma was documented in 19 patients (12.3%), and wound-healing disturbance occurred in 16 patients (10.4%). All patients with hematoma or wound-healing disturbance underwent revision without exchange or modification of the implant components.

**Fig. 5:**
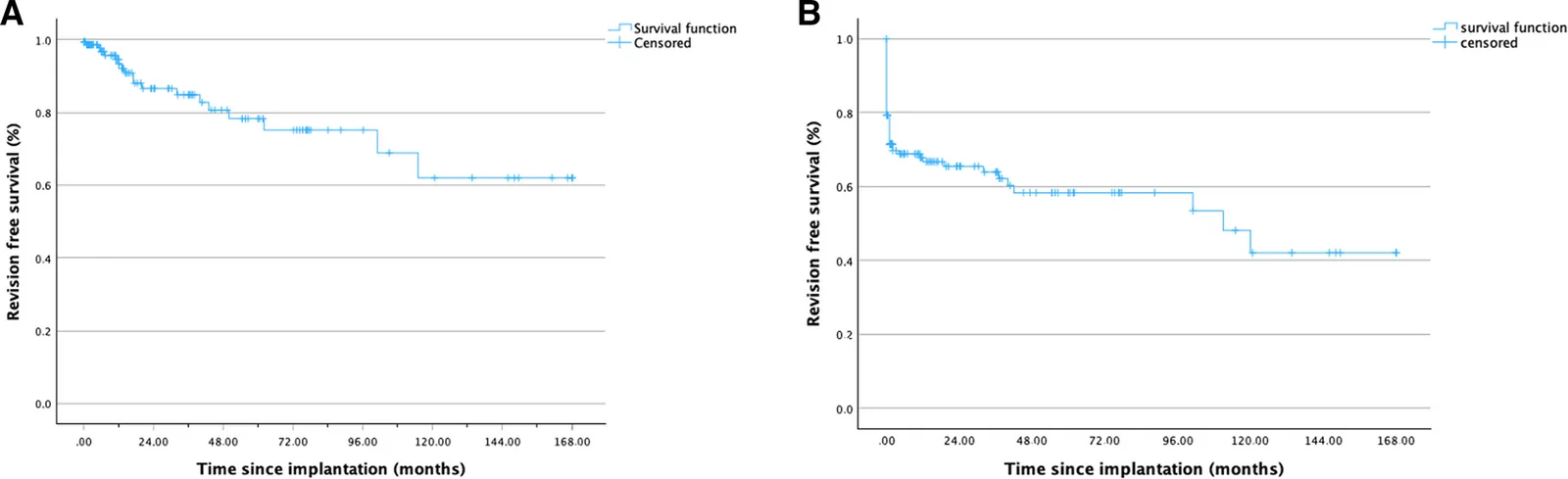
**A** Kaplan–Meier survival analysis of implant survivorship with explantation as the endpoint. **B** Kaplan–Meier survival analysis of implant survivorship with survival free from reoperation for any reason

Kaplan–Meier analysis estimated implant survivorship of 0.85 (SE = 0.04) at 3 years and 0.78 (SE = 0.05) at 5 years, with explantation defined as the endpoint. At the latest available follow-up of 168 months, implant survivorship was 0.62 (SE = 0.10). Patients without explantation were censored at their individual last follow-up (Fig. 5A). The number of patients at risk declined markedly over time: 27 were at risk at 60 months and 8 at 120 months.

Estimated revision-free survival (reoperation for any reason) was 0.64 (SE = 0.04) at 3 years, 0.58 (SE = 0.05) at 5 years, and 0.42 (SE = 0.09) at the latest follow-up of 168 months (Fig. 5B). Any reoperation, regardless of cause, was treated as the endpoint event, and patients without reoperation were censored at their last follow-up. The number at risk fell from 21 at 60 months to 7 at 120 months. Flange design comprised monoflange components in 101 patients (65.6%), biflange components in 44 (28.6%), and triflange components in 9 (5.8%). For the multivariable model, flange design was entered as a binary covariate contrasting monoflange (*n* = 101) with multiflange constructs (*n* = 53). An exploratory multivariable Cox proportional-hazards model for implant removal included infection status, pelvic discontinuity, flange design, and age at surgery. Flange design was the only covariate significantly associated with explantation (HR 17.70, 95% CI 4.78–65.52; *p* < 0.001), pelvic discontinuity (HR 2.69, 95% CI 0.73–9.92; *p* = 0.14), infection status (HR 1.01, 95% CI 0.35–2.97; *p* = 0.98), and age at surgery (HR 0.99 per year, 95% CI 0.96–1.03; *p* = 0.76) were not.

**Fig. 6:**
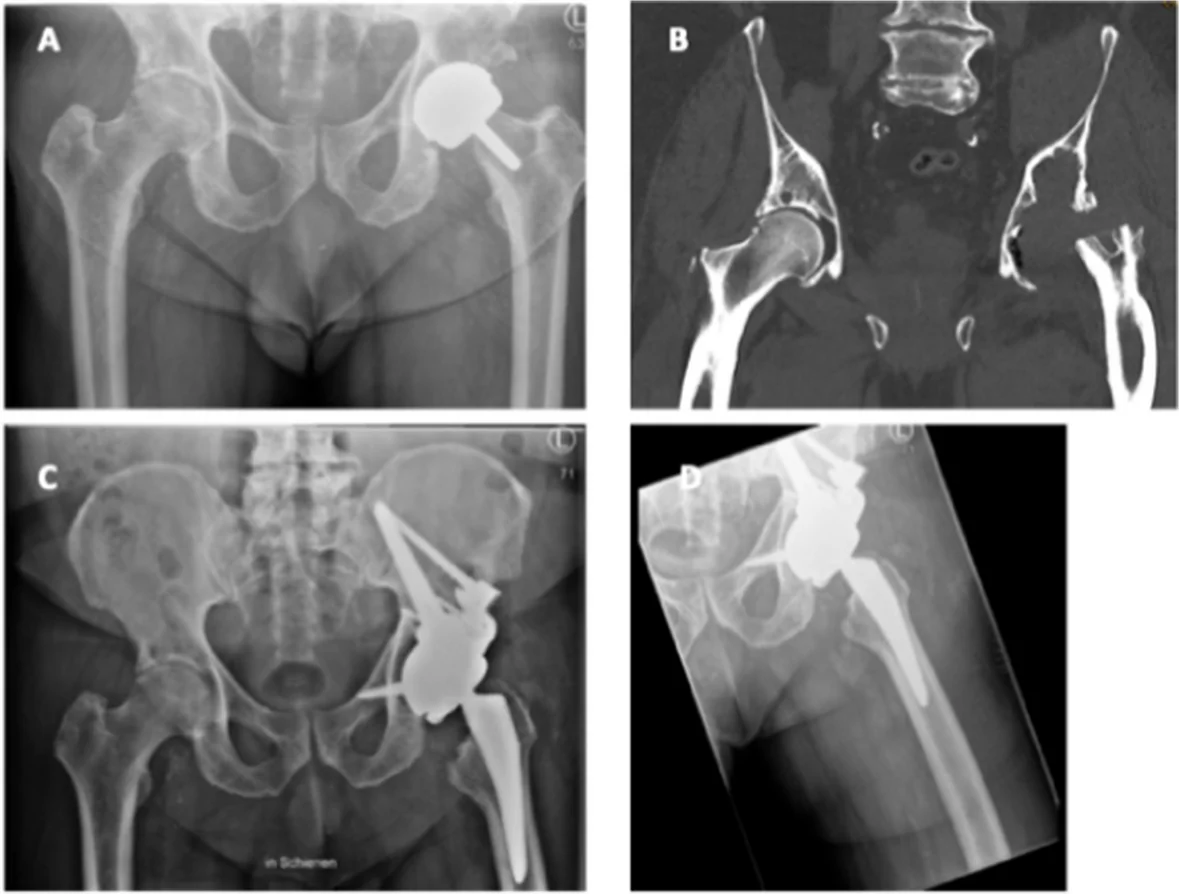
CMAC in extensive cranial osteolysis: imaging follow-up. **A** Preoperative radiograph demonstrating a loosened McMinn prosthesis on the left side with an associated extensive pseudotumor. **B** Post-explantation CT imaging showing postoperative conditions with marked metallosis-related granulomatous tissue and extensive destruction of the ilium, particularly involving the acetabular dome, corresponding to ADC type 3C. **C** Postoperative radiographic evaluation 12 months after implantation of the custom-made acetabular implant, demonstrating stable component positioning and satisfactory osseointegration during follow-up. **D** Lauenstein view at 12 months showing stable positioning without radiographic signs of loosening

Radiographic evaluation identified screw breakage in 5 (3.4%) patients during follow-up. Screw malposition requiring revision surgery occurred in 2 (1.4%) patients. Radiographs were incomplete in 8 patients, leaving 146 implants available for radiographic assessment. Applying the criteria of Moore et al. [18], radiographic osseointegration (≥ 3 of 5 signs) was present in 132 of 146 reconstructions (90.4%) at a minimum of twelve months postoperatively. Radiographic failure was defined as migration > 5 mm, inclination change > 5°, progressive radiolucent lines, or hardware fracture. The remaining 14 reconstructions (9.6%) showed signs of radiographic loosening, of which 6 (4.1%) were of septic and 8 (5.5%) of aseptic origin (Fig. 6).

## Discussion

This study presents the largest single-center experience with custom-made acetabular implants over 18 years, providing valuable mid-term insights into complication rates, implant evolution, and clinical outcomes in a highly complex patient population. The most frequent adverse events observed were postoperative periprosthetic infection and dislocation, consistent with previous reports [19–21]. In the overall cohort, 90.4% of CMAC met the radiographic osseointegration criteria, indicating reliable bony integration of the implant.

Over the 18-year observation period, our CMAC design evolved substantially from early triflange constructs to monoflange configurations with locking iliac fixation, macroporous surfaces, and optional transiliosacral screw stabilization for pelvic discontinuity. Satisfactory mid-term survivorship has been reported following revision total hip arthroplasty using custom-made triflange acetabular components [22]. However, the large implant size and extensive surgical exposure required were associated with increased soft-tissue trauma and a higher risk of neurovascular injury, particularly to the superior gluteal nerve during dissection and implantation [23, 24]. These factors may contribute to higher rates of postoperative infection and dislocation, emphasizing the limitations of the triflange design and the need for less invasive alternatives such as monoflange systems. Our findings align with recent studies by Schlossmacher et al. and Gehrke et al., which have demonstrated that precise 3D preoperative planning and individualized implant geometry enable accurate restoration of the hip center of rotation and load transfer to viable host bone, thereby improving functional outcomes [25, 26].

For cases with pelvic discontinuity, our current custom design increasingly incorporates trans-iliosacral screws. In particular, Paprosky type 3B and ADC type 4 defects are associated with extensive osteolysis involving the os ischium and os pubis, resulting in compromised bone quality and limited fixation potential. Traditional reconstructive concepts have therefore focused on anatomical restoration of pelvic ring continuity through inferior bridging constructs or discontinuity-spanning fixation [27, 28]. However, these strategies are frequently constrained by compromised bone quality and may therefore be associated with increased surgical complexity, limited fixation reliability, and higher complication rates. In contrast, other studies have demonstrated that reliable outcomes can be achieved with custom-made components by prioritizing stable fixation to the remaining pelvic bone, particularly the ilium, without requiring consistent anatomical closure of the discontinuity [29, 30]. In line with these observations, our evolving treatment philosophy focuses on achieving durable cranial and iliosacral fixation rather than anatomical discontinuity closure, allowing reliable reconstruction even in advanced defects. This should be interpreted in the context of alternative strategies, particularly modular porous-metal augment and cup–cage constructs, which have shown reliable mid-term osseointegration, functional improvement, and acceptable complication rates [31]. These data derive predominantly from Paprosky 2–3 defects, whereas our cohort comprises Paprosky 3A/3B defects, limiting direct comparison. CMAC and cup–cage constructs are therefore best regarded as complementary, defect-adapted options rather than directly comparable alternatives.

Continuous technological refinement parallels the broader trend seen in modern revision arthroplasty, where individualized 3D-printed components are increasingly recognized as the new standard for addressing severe acetabular bone defects [3, 32]. Consistent with our cumulative clinical experience, recent studies have shown that custom-made acetabular implants enable accurate restoration of hip anatomy and implant positioning, even in cases with severe acetabular bone loss. These findings highlight the growing role of patient-specific, 3D-printed solutions in complex revision hip arthroplasty [25, 33].

Nevertheless, complications such as dislocation and postoperative periprosthetic joint infection remain relevant challenges. In our cohort, postoperative periprosthetic joint infection (11.0%) and dislocation (10.4%) were the most frequent complications, broadly comparable to other series [20, 34]. The mean improvement of 9.23 points in the OHS exceeds the minimal important change of 8.6 points reported for revision hip replacement [35], and can therefore be regarded as clinically meaningful, in line with comparable series [19]. Cross-study comparison remains limited, as different outcome instruments with differing scales are used throughout the literature.

While soft-tissue quality and prior surgical history are major contributing factors, future strategies must also focus on surface modifications and antimicrobial technologies. The incorporation of antimicrobial coatings or ion-releasing titanium alloys may reduce bacterial colonization on implant surfaces and thus lower the risk of reinfection in large-scale reconstructions, a direction already explored in recent biomaterial research [36, 37]. A practical limitation is the lead time and cost of manufacturing: from CT imaging and design to surgical implantation, weeks may pass, during which the defect can evolve, or further bone resorption may occur.

Several limitations warrant emphasis. First, the design was retrospective and without a control group, which reflects the nature of its indication, as CMAC are used when standard reconstruction is impossible, the only alternatives being a permanent Girdlestone situation or an untreated defect. Second, over the 18 years, surgical technique, additive-manufacturing processes, bearing selection, perioperative and antimicrobial protocols, and the implant manufacturer all changed. These factors are inseparably confounded with implant design and could not be adjusted for, although all implants followed our institutional specifications and the same philosophy, limiting between-system heterogeneity. Third, follow-up was right-skewed, so late survivorship estimates rest on few patients at risk. Finally, the small number of events constrained multivariable modelling. The exploratory Cox regression is prone to overfitting and should be regarded as hypothesis-generating, with the stratified analysis remaining our primary approach. In addition, patients undergoing re-implantation after explantation are not re-entered into the survivorship analysis. While this avoids informative selection, it means that outcomes after re-implantation are not captured by the survival estimates here.

## Conclusion

This single-center study, encompassing 154 patients treated with custom-made acetabular implants, demonstrates that individualized reconstruction provides a reliable solution for extensive acetabular bone defects at mid-term follow-up. The findings support patient-specific implant design, while underscoring that instability and postoperative periprosthetic infection remain important challenges. Whether the evolution from multiflange to monoflange cranial-dominant fixation translates into superior outcomes remains to be demonstrated and will require dedicated comparative studies.

## Data Availability

The datasets generated and/or analyzed during the current study are not publicly available due to patient privacy regulations under the General Data Protection Regulation (GDPR) and institutional data protection policies of the University Hospital Bonn. Still, they are available from the corresponding author on reasonable request and with permission from the Institutional Review Board.
